# Whole Genome Sequencing and Annotation of *Naematelia aurantialba* (Basidiomycota, Edible-Medicinal Fungi)

**DOI:** 10.3390/jof8010006

**Published:** 2021-12-22

**Authors:** Tao Sun, Yixuan Zhang, Hao Jiang, Kai Yang, Shiyu Wang, Rui Wang, Sha Li, Peng Lei, Hong Xu, Yibin Qiu, Dafeng Sun

**Affiliations:** 1 State Key Laboratory of Materials-Oriented Chemical Engineering, College of Food Science and Light Industry, Nanjing Tech University, Nanjing 211816, China; sun-tao@njtech.edu.cn (T.S.); zhangyixuan@njtech.edu.cn (Y.Z.); jianghao@njtech.edu.cn (H.J.); 201961119005@njtech.edu.cn (K.Y.); 201921019081@njtech.edu.cn (S.W.); ruiwang2013@njtech.edu.cn (R.W.); lisha@njtech.edu.cn (S.L.); 2 College of Light Industry and Food Engineering, Nanjing Forestry University, Nanjing 210037, China; qyb@njfu.edu.cn; 3 Kunming Edible Fungi Institute of All China Federation of Supply and Marketing Cooperatives, Kunming 650032, China; sdafeng@163.com

**Keywords:** *Naematelia aurantialba*, whole-genome sequencing, functional annotation, secondary metabolism, polysaccharides

## Abstract

*Naematelia aurantialba* is a rare edible fungus with both nutritional and medicinal values and especially rich in bioactive polysaccharides. However, due to the lack of genomic information, researches on the mining of active compounds, artificial breeding and cultivation, genetics, and molecular biology are limited. To facilitate the medicinal and food applications of *N. aurantialba*, we sequenced and analyzed the whole genome of *N. aurantialba* for the first time. The 21-Mb genome contained 15 contigs, and a total of 5860 protein-coding genes were predicted. The genome sequence shows that 296 genes are related to polysaccharide synthesis, including 15 genes related to nucleoside-activated sugar synthesis and 11 genes related to glucan synthesis. The genome also contains genes and gene clusters for the synthesis of other active substances, including terpenoids, unsaturated fatty acids, and bioactive proteins. In addition, it was also found that *N. aurantialba* was more closely related to *Naematelia encephala* than to *Tremella fuciformis.* In short, this study provides a reference for molecular cognition of *N. aurantialba* and related researches.

## 1. Introduction

Mushrooms are widely distributed food and medicine resource on Earth and have excellent nutritional and medicinal value [1,2]. The mushrooms are considered as superfoods, which are among the world’s healthiest foods, and approximately 50% of edible mushrooms are recognized as functional foods [3]. *Naematelia aurantialba* syn. *Tremella aurantialba*, also known as Jin’er, an edible and medicinal fungus distributed in China, is widely popular because of its unique flavor and high nutritional value in its fruiting bodies [4]. Previous studies have reported that the main medicinal functions of *N. aurantialba* include antioxidant, anti-inflammatory, anti-tumor, and immunomodulatory effects, for which polysaccharides, active proteins, and terpenoids are responsible [5,6,7,8,9]. Polysaccharides are recognized as one of the most active compounds in *N. aurantialba*, which has a total carbohydrate content of 74.11%, including a 40% content of water-soluble polysaccharides [7]. In addition, *N. aurantialba* is a fungus containing phenolic acids and flavonoids, which has antioxidant effects [10]. The fruiting body of *N. aurantialba* grows on rotten wood, which has the ability to degrade lignocellulose because it is rich in carbohydrate-active enzymes (CAZymes) [11,12]. It is also possible that *N. aurantialba* has these degrading enzymes, and the activities of these enzymes may be beneficial to biomass utilization and organic pollutant degradation.

With the rapid development of DNA sequencing technology and gene-editing technology, strengthening the polysaccharide synthetic pathway through metabolic engineering strategies has become a possible way to improve the yield of mushroom polysaccharides, which can lead to the industrial production of polysaccharides in the future [13,14,15,16]. However, there have been no reports on improving the production of *N. aurantialba* polysaccharides by genetic modification techniques. The reason is mostly due to the lack of relevant genome-wide information, which limits the development of genetic manipulation methods. In addition, the development of genome sequencing technologies has provided new insights into active compound mining, variety breeding, high-yield cultivation, and population genetics [17,18,19,20,21]. The taxonomic boundaries between mushrooms are blurred, and fungal names have long been controversial, which has led to slow development of good quality varieties of mushrooms and thus difficulties in achieving large-scale production [22].

The medicinally valuable sang’huang recorded in the ancient book of traditional Chinese medicine has previously been considered as *Sanghuangporus baumii* and *Sanghuangporus vaninii*; yet, Ying et al. clarified its taxonomic status by comparative genomic analysis and named it *sanghuangporus sangguang* [22,23]. The golden needling mushroom in East Asia has been reported as Asian *Flammulina velutipes* or *Flammulina velutipes var. filiformis* [24]. However, the phylogenetic results based on multiple gene markers and morphological comparisons suggest that so-called *F. velutipes* in East Asia, unlike the European winter mushroom *F. velutipes*, should be treated as a separate species, namely *F. filiformis* [25]. A similar problem was reported for Jin’er, which was previously reported as *Tremella mesenterica* [26]. Bandoni R.J. studied the morphological features of Jin’er and named it *T. aurantialba* [11]. Until 2015, Liu et al. investigated the phylogenetic relationship of Tremellomycetes by phylogenetic trees constructed by seven gene sequences, eventually naming them *N. aurantialba* [27]. Therefore, it is necessary to further clarify the taxonomic status of *N. aurantialba* genetically from the population level.

In recent years, the genomes of some basidiomycetes have been obtained, including *Agaricus bisporus* [28], *Auricularia heimuer* [17], *Coprinopsis cinerea* [29], *G. lucidum* [30], *Hericium erinaceus* [21], *Lentinula edodes* [31], *Naematelia encephala* [32], *Tremella fuciformis* [33], and *T. mesenterica* [34]. The availability of these increased genome sequences has promoted research on gene diversity and the identification of genes involved in the biosynthesis of secondary metabolites through genome mining. Although *N. aurantialba* has many important characteristics, there are only about 13 available nucleotide sequences for *N. aurantialba* in the National Center for Biotechnology Information (NCBI) database, most of which are used for phylogenetic analysis. Therefore, the current genetic sequence resources are not enough to reveal the pharmacological mechanism of *N. aurantialba* at the molecular level.

Therefore, in this study, we aimed to introduce the whole genome sequence of *N. aurantialba* NX-20 and to elucidate the its genome through comparison with the genomes of 18 basidiomycetes. We also aimed to investigate functional annotations (Gene Ontology (GO), Kyoto Encyclopedia of Genes and Genomes (KEGG), Clusters of Orthologous Groups (KOG), Transporter Classification Database (TCDB), etc.) to predict the genes or gene clusters involved in the biosynthesis of polysaccharides and other secondary metabolites.

## 2. Materials and Methods

### 2.1. Fungal Strains and Strain Culture

**Figure 1:**
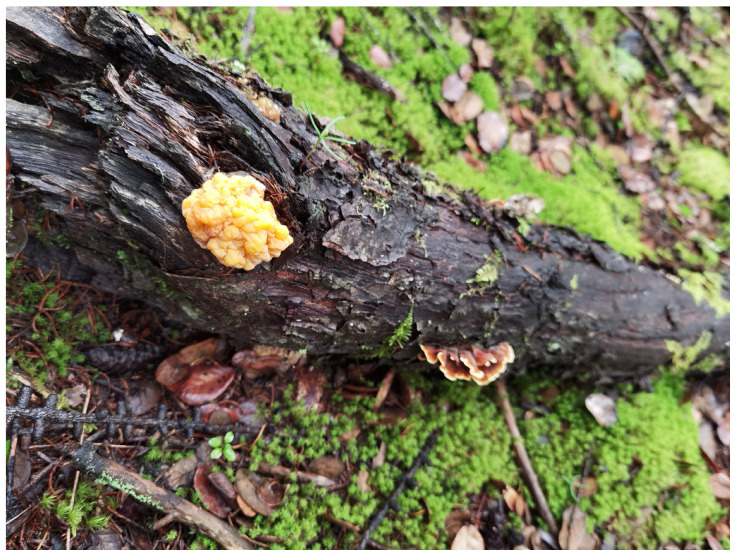
Fruiting bodies of *N. aurantialba*.

The fruiting bodies of *N. aurantialba* were collected from Kunming, Yunnan Province, China (Figure 1). A single spore strain was obtained from the fruiting body by the spore ejection method, and the strain was identified as *N. aurantialba*, which we named *N. aurantialba* NX-20 [35]. At present, this strain has been preserved in the China General Microbiological Culture Collection Center (CGMCC 18588). To obtain sufficient cell amounts for genomic DNA extraction, *N. aurantialba* NX-20 was inoculated into potato dextrose broth medium and grown at 25 °C with constant shaking (200 rpm) for 3 d [35].

### 2.2. Extraction of Genome DNA

After fermentation, the spore cells were collected by centrifugation at 8000× *g* for 5 min, and sterile water (three rinses) was used to remove the medium and metabolites attached to the spore cell surface. The sodium dodecyl sulfate (SDS) method was used to extract the genomic DNA, and agarose gel electrophoresis was performed to check its integrity [23].

### 2.3. De Novo Sequencing and Genome Assembly

#### 2.3.1. De Novo Sequencing

The 20-kb SMRTbell library was constructed using the SMRTbell TM Template Prep Kit (version 1.0) [36]. The 350-bp small, fragmented library was constructed using the NEBNext^®^ Ultra ^TM^ DNA Library Prep Kit (NEB, Ipswich, MA, USA) [37]. After the library was qualified, the whole genome of *N. aurantialba* NX-20 was sequenced using the PacBio Sequel platform and Illumina NovaSeq PE150 at the Beijing Novo Gene Bioinformatics Technology Co., Ltd. (Beijing, China) [38].

#### 2.3.2. Genome Assembly and Assessment

Regarding the Illumina NovaSeq PE150 platform, firstly, SOAP denovo (version 2.04), SPAdes (version 3.1.1), and ABySS (version 2.0.2) assembly software were used to assemble the preprocessed clean data, and CISA (version 1.3) software was used for integration [39,40,41,42]. Second, GapCloser (version: 1.12) software was used to optimize the preliminary assembly results and fill holes so as to obtain the final assembly results [39]. Finally, the fragments below 500 bp were filtered out, and the contaminated samples were decontaminated again, evaluated, statistically analyzed, and subsequently used for gene prediction.

Regarding the PacBio Sequel platform, on the basis of removing the low-quality reads (less than 500 bp) from the raw data, the automatic error correction function of the SMRT portal software was used to further improve the accuracy of the seed sequences, and finally, the variant caller module of the SMRT link v5.0.1 software was used to correct and count the variant sites in the initial assembly results using the arrow algorithm [43]. Benchmarking Universal Single-Copy Orthologs (BUSCO) v 3.0.2 software was used to assess the completeness of the genome assembly and single-copy ortholog annotation [44]. The lineage dataset of BUSCO was fungi_odb9 (creation date: 13 February 2016; number of species: 85; number of BUSCOs: 290). In addition, the assembly of *N. aurantialba* was compared with that of *T. fuciformis*, *T. mesenterica*, and *N. encephala*.

### 2.4. Genome Component Prezdiction

Genome component predictions were divided into predictions for coding genes, repetitive sequences, and noncoding RNAs. First, gene prediction was a combination of de-novo prediction and homology prediction, Augustus version 3.3.3 was used to de-novo predict protein coding gene models, and genomic information of *N. encephala* was used to homology predict protein coding gene models [45]. Then, the scattered repeats were predicted using RepeatMasker software (version 4.0.5), and tandem repeats finder (TRF, version 4.07b) was used to search for tandem repeats in the DNA sequences [46,47]. Finally, based on the combination of the RNA library, tRNAscan-SE software (version 1.3.1), rRNAmmer software (version 1.2), and Rfam database (version 9.1) were used to predict the structure of tRNA, rRNA, and sRNA [48,49,50].

### 2.5. Genome Annotation

Genomic functional annotation mainly involved BLAST alignment of the predicted genes from *N. aurantialba* against various functional databases, namely Gene Ontology, KEGG, KOG, Non-Redundant Protein Database (NR) databases, Transporter Classification Database (TCDB), Carbohydrate-Active enzymes (CAZymes), P450, and Swiss-Prot. The E-value was less than 1 × 10^−5^, and the minimal alignment length percentage was larger than 40%.

SignalP (version 4.1) and antiSMASH (version 6.0) software were used to predict the secretory proteins and secondary metabolic gene clusters in the *N. aurantialba* genome, respectively [51,52].

### 2.6. Comparative Genomics Analysis

#### 2.6.1. Core-Pan Genome, Phylogenetic, and Gene Family Analysis

Core-pan genome were analyzed by the Cluster Database at High Identity with Tolerance (CD-HIT) rapid clustering of similar proteins software with a threshold of 50% pairwise identity and 0.7 length difference cutoff in amino acid [53]. TreeBeST or PhyML was adopted to construct the developmental evolutionary tree based on Muscle, and the bootstrap was set to 1000 with homologous genes [54].

Using several softwares, the gene family of *N. aurantialba* and nine other fungi was constructed: First, Blast (Version 2.2.26) was used to pairwise align all genes, after which Solar (Version 0.9.6) was used to remove redundancy, and Hcluster_sg (version 0.5.0) was used to perform gene family clustering based on the alignment results [55].

#### 2.6.2. Genomic Synteny

MUMmer and LASTZ tools were used for genomic alignment, followed by genomic commonality analysis based on the alignment results [56,57].

### 2.7. Other Basidiomycete Genome Sources

The whole genome sequences of other Basidiomycetes used in the present study were downloaded from the NCBI (National Center for Biotechnology Information, https://www.ncbi.nlm.nih.gov/genome, accessed on: 2 September 2021) Whole Genome Shotgun (WGS) database, and the U.S. Department of Energy Joint Genome Institute website (http://genome.jgi.doe.gov/, accessed on: 2 September 2021) (Table S1).

## 3. Results and Discussion

### 3.1. Sequencing and Assembly Data

The final genome was composed of 15 contigs after genome assembly, correction, and optimization. The total length of all assembled contigs was 20,998,359 bp with a GC content of 56.42%, encoding 5860 genes with an N50 value of 1,814,705 bp. The maximum contig length among the assembled sequences was 2,546,384 bp, and the details of data generation are listed in Table 1.

### 3.2. Genomic Features

To date, four genomes (*Tremella fuciformis* tr26, *Tremella mesenterica* DSM 1558, *Tremella mesenterica* ATCC 28783, and *Naematelia encephala* 68-887.2) belonging to the Tremellales, an edible mushroom, are available in the NCBI WGS database. We found that *N. aurantialba* has a guanosine-cytosine (GC)% similar to that of *T. fuciformis* tr26 but has a difference in length. *N. aurantialba* has a genomic length similar to that of N. encephala, but the GC% is significantly different. It is striking that *N. aurantialba* differs from *T. mesenterica* in terms of both genome length and GC%. In short, *N. aurantialba* has diverged somewhat from several other genomic information, which is perhaps because the whole-genome sequencing values of other genomes were obtained with only second generation sequencing technology, thereby leading to less complete genomic data, whereas our results were more precise in the way that third-generation sequencing generation sequencing combined with second-generation sequencing was used [58]. The details of the assembly summary statistics are presented in Table 2.

As shown in Table 2, the completeness of the genome was 93.1%, and the fragmentation rate was 2.4%, indicating that the completeness of the genome assembly and annotation indicated that the annotation set was complete.

### 3.3. Repeat Sequence

The repeat sequence data of *N. aurantialba* NX-20 are shown in Table 3 and were subdivided into interspersed repeats (IR) and tandem repeats (TR), in which long IRs and TR units have transposition activity and species composition specificity, respectively.

The total length of repetitive sequences was 774,977 bp, which accounted for 3.6902% of the genome length. A total of 1549 IR, 191,748 bp in length, accounted for 0.9132% of the genome length. The short interspersed nuclear elements (SINEs) and long interspersed nuclear elements (LINEs) accounted for 0.0049% and 0.1883% of the assembled genome, respectively, in the IR. TR represented 2.7775% of the assembled genomes. The Microsatellite DNA and Minisatellite DNA accounted for 0.4353% and 2.1576% of the assembled genome, respectively, in the TR. In comparison to the other three fungi’s repeated sequence predictions (Table S2), *N. aurantialba* had more genes in the LINE in IR as well as Minisatellite DNA and Microsatellite DNA in TR. Microsatellite DNA can be found in both the protein-coding and noncoding sections of genes, and it plays a role in gene control, phenotypic diversity, and evolution [59]. Because it is highly polymorphic, inherited in a codominant form, and widely scattered throughout the genome, it is regarded suitable for constructing PCR-based markers in genetic investigations [60]. As a result, the findings can serve as a theoretical foundation for the development of microsatellite markers in *N. aurantialba*.

### 3.4. Noncoding RNA

Noncoding RNAs (ncRNAs), a class of RNA molecules that performs a variety of biological functions and does not carry information into proteins, directly exerts its effects on life activities at the RNA level. The results of noncoding RNAs in the *N. aurantialba* genome are shown in Table 4. With regard to RNA, 44 tRNAs, 11 rRNAs, and seven snRNAs were predicted. Of the tRNAs, one may be a pseudogene, and the 96 anticodon tRNAs correspond to 19 common amino acid codons. Among the rRNAs, there are 9 5s_rRNAs, 1 18s_rRNAs, and one 28s_rRNA. In addition, there are no miRNAs in this genome assembly because there is currently no basidiomycetes miRNA database [61]. Table S2 shows that the ncRNAs-related genes of the four edible mushrooms have poor differential conservation, which might be due to the fact that majority of the ncRNAs found in fungi of the genus mushroom have no homologs in other fungal groupings [61]. There were no snRNA encoding genes in the other three edible Tremellales fungus, but *N. aurantialba* had seven snRNA-related genes. The reason of this phenomena has to be investigated further.

### 3.5. Gene Function Annotation

To predict the protein sequences, a similarity analysis of 5860 non-redundant genes in multiple public databases (GO, KEGG, KOG, NR, TCDB, Pfam, CAZy, P450, Swiss-Prot, SignalP, TMHMM, PHI, and DFVF) identified 5488 genes that were annotated, which accounted for 93.65% of the assembled genome. The annotation results are shown in Table S3 and Figure S1.

#### 3.5.1. KOG Annotations

The KOG database is a database of orthologs for eukaryotes belonging to the COG database [62]. A statistical map of the number of annotated genes in the KOG database is shown in Figure S2. A total of 1495 genes were assigned to 24 categories of KOG, of which the top four were “Posttranslational modification, protein turnover, chaperones” (184, 12.31%), “Translation, ribosomal structure, and biogenesis” (182, 12.17%),“General function prediction only” (157, 10.50%), and “Energy production and conversion” (122, 8.16%). *N. aurantialba* has more genes in “Lipid transport and metabolism”, “Translation, ribosomal structure and biogenesis”, and “Cytoskeleton” compared to the KOG annotations of the other three edible fungi (Figure S3 and Table S4). To some extent, fatty acid types can be used to distinguish Tremellales fungi types; however, strains of the same species have similar long-chain fatty acid compositions, which is due to differences in fatty acid synthetic capacity among fungi, with the number of genes being one of the most important factors affecting fatty acid synthetic capacity [63,64]. As a result, our findings suggest that *N. aurantialba* has a greater capability for fatty acid production than the other three fungi.

#### 3.5.2. GO Annotations

According to the GO database, 3858 genes were assigned to three major categories: biological processes (24 branches), cellular components (11 branches), and molecular functions (11 branches). These were mainly distributed in four functional entries, “cellular process”, “metabolic process”, “binding”, and “catalytic activity”, of which the number of annotated genes was 2153, 1990, 1940, and 1774, respectively (Figure S4). *N. aurantialba* had more genes in common subcategories of “developmental process”, “immune system process”, “negative regulation of biological process”, “reproduction”, “rhythmic process”, and “reproductive process” within the biological process categories and “structural molecule activity” within the molecular function categories when compared to the GO annotations of the other three fungi (Table S4). However, the number of GO functions associated with “metabolic process” was lower than in the other three fungi. The cause of this phenomena is still being investigated.

#### 3.5.3. KEGG Annotations

To further systematically analyze the metabolic pathways of gene products in cells and the functions of these gene products, the KEGG database was used to annotate the gene functions of *N. aurantialba*. A statistical map of the number of annotated genes in the KEGG database is shown in Figure S5. Four thousand six hundred and four genes were assigned to six major categories in KEGG: cellular processes (five branches, 418, 9.08%), environmental information processing (three branches, 208, 4.52%), genetic information (four branches, 657, 14.27%), human diseases (11 branches, 615, 13.36%), large metabolism (12 branches, 1670, 36.27%), and organismal systems (10 branches, 466, 10.12%). *N. aurantialba* possesses more genes in carbohydrate metabolism than the other three fungi (Table S4), including “ascorbate and aldehyde metabolism”, “citrate cycle (TCA cycle)”, “inositol phosphate metabolism”, and “propanoate metabolism”. The findings also suggested that *N. aurantialba* had a high capacity for polysaccharide production.

#### 3.5.4. CAZymes

**Figure 2:**
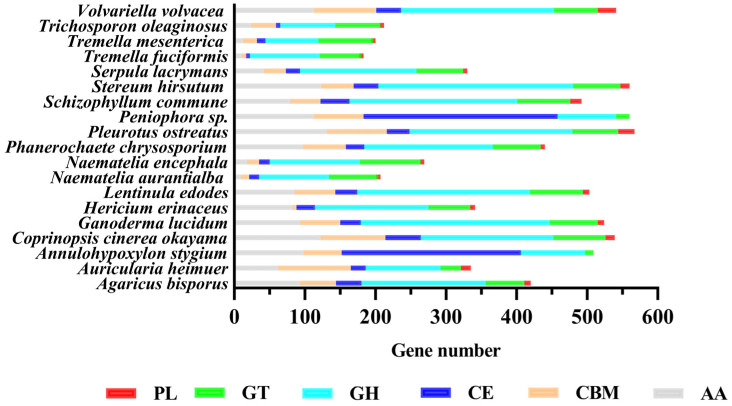
The number of CAZymes genes in *N. aurantialba* and the other 18 fungi.

In this study, the CAZy database was used to map the genome of *N. aurantialba* to study the distribution of CAZymes. A total of 207 genes were annotated as CAZymes family in this study, including 99 glycoside hydrolases (GHs), 70 glycosyl transferases (GTs), three polysaccharide lyases (PLs), 14 carbohydrate esterases (CEs), 12 carbohydrate-binding modules (CBMs), and nine auxiliary activities (AAs) (Figure 2 and Table S5).

In nature, the fruiting body of *N. aurantialba* usually grows on dead wood as a type of wood rot fungus, so it has a strong ability for lignin fiber degradation [41]. The CAZyme spectra were compared between *N. aurantialba* and 18 other Basidiomycete species. Although the number of CAZymes genes annotated by *N. aurantialba* was close to that of the other four fungi of the Tremellales, compared with the other 14 species of white rot fungi, the contents of AAs, GHs, CBM, and PL in *N. aurantialba* were much lower than their average values (Figure 2 and Table S5). This may be because *N. aurantialba* is a parasitic fungus with *Stereum hirsutum* as its host. *S. hirsutum* is rich in CAZymes (560 genes), and with its help, *N. aurantialba* is able to utilize plant cell wall polysaccharides, such as cellulose and hemicellulose. This phenomenon is very common in fungi of the Tremellales, which live parasitic lives and can utilize plant polysaccharides for growth only with the help of host fungi [65], for example, *T. fuciformis* (CAZymes,183 genes)-parasitized *Annulohypoxylon stygium* (CAZymes, 541 genes) and *T. mesenterica* (CAZymes, 200 genes)-parasitized *Peniophora* sp. (CAZymes, 593 genes). In brief, we speculated that a limited number of CAZymes could protect the host cell wall from massive destruction by parasitic fungi. The CAZyme gene annotation of *N. aurantialba* confirmed the suitability of the enzyme repertoire of this class of fungal species for parasitism and revealed strategies for host interactions with parasitic organisms (Table S5) [65].

In terms of quantity, the number of CAZyme genes associated with cellulases, hemicellulases, and pectinases in the *N. aurantialba* genome were 33, 55, and 17, respectively. However, the number of genes contained by its host counterpart *S. hirsutum* was 132, 144, and 81.

Compared with the abundant plant cell wall polysaccharide-degrading enzymes of *S. hirsutum*, *N. aurantialba* has almost no oxidoreductase (AA3, AA8, and AA9), cellulose-degrading enzymes (GH6, GH7, GH12, and GH44), hemicellulose-degrading enzymes (GH10, GH11, GH12, GH27, GH35, GH74, GH93, and GH95), and pectinase (GH93, PL1, PL3, and PL4). It was shown that *N. aurantialba* has a low number of genes identified in the genome to degrade plant cell wall polysaccharides (cellulose, hemicellulose, and pectin), whereas *S. hirsutum* has a strong ability to disintegrate. Hence, we speculated that *S. hirsutum* hydrolyzed plant cell polysaccharides into cellobiose or glucose for the development and growth of *N. aurantialba* during cultivation [66].

The CAZyme annotation can provide a reference not only for the analysis of polysaccharide-degrading enzyme lines but also for the analysis of polysaccharide synthetic capacity. A total of 35 genes related to the synthesis of fungal cell walls (chitin and glucan) were identified (Table S5).

#### 3.5.5. The Cytochromes P450 (CYPs) Family

The cytochrome P450s (CYP450) family is a superfamily of ferrous heme thiolate proteins that are involved in physiological processes, including detoxification, xenobiotic degradation, and biosynthesis of secondary metabolites [67]. The KEGG analysis showed that *N. aurantialba* has four and four genes in “metabolism of xenobiotics by cytochrome P450” and “drug metabolism—cytochrome P450”, respectively (Table S6). For further analysis, the CYP family of *N. aurantialba* was predicted using the databases (Table S6). The results showed that *N. aurantialba* contains 26 genes, with only four class CYPs, which is much lower than that of wood rot fungi, such as *S. hirsutum* (536 genes). Interestingly, Akapo et al. found that *T. mesenterica* (eight genes) and *N. encephala* (10 genes) of the Tremellales had lower numbers of CYPs [65].

This phenomenon was probably attributed to the parasitic lifestyle of fungi in the Tremellales, whose ecological niches are rich in simple-source organic nutrients, losing a considerable amount during long-term adaptation to the host-derived simple-carbon-source CYPs, thereby compressing genome size [65,68]. Intriguingly, the same phenomenon has been observed in fungal species belonging to the subphylum Saccharomycotina, where the niche is highly enriched in simple organic nutrients [69].

### 3.6. Secondary Metabolites

In the fields of modern food nutrition and pharmacology, mushrooms have attracted much interest because of their abundant secondary metabolites, which have been shown to possess various bioactive pharmacological properties, such as immunomodulatory, anti-inflammatory, anti-aging, antioxidant, and antitumor [70]. A total of 215 classes of enzymes involved in “biosynthesis of secondary metabolites” (KO 01110) were predicted, as shown in Table S7.

As shown in Table S8, five gene clusters (45 genes) potentially involved in secondary metabolite biosynthesis were predicted. The predicted gene cluster included one betalactone, two NRPS-like, and two terpenes. No PKS synthesis genes were found in *N. aurantialba*, which was consistent with most Basidiomycetes. Saponin was extracted from *N. aurantialba* using a hot water extraction technique, which had a better hypolipidemic impact [71]. The phenolic and flavonoid of *N. aurantialba* was extracted using an organic solvent extraction technique, which revealed strong antioxidant activity [10,72]. Therefore, this finding suggests that *N. aurantialba* has the potential to synthesize biologically active secondary metabolites.

In fungi, terpenes are a class of identified secondary metabolites with potent biological activities, which are usually derived from dimethylallyl diphosphate (DMAPP) and isopentenyl diphosphate (IPP), produced by acetyl coenzyme A (acetyl-CoA) via the mevalonate pathway. In this study, a total of 13 classes of enzymes involved in “terpenoid backbone biosynthesis” were identified, which generated DMAPP and IPP from acetyl CoA via the mevalonate pathway.

Like most Basidiomycetes, *N. aurantialba* had few genes of the 1-deoxy-D-xylulose 5-phosphate/2-C-methyl-D-erythritol 4-phosphate (MEP/DOXP) pathway but was enriched with genes of the DMAPP/IPP pathway (Table S8 and Figure S6) [73]. Moreover, there were a total of six classes of enzymes in the “ubiquinone and other terpenoid quinone biosynthesis” pathways, indicating that *N. aurantialba* may has the ability to synthesize ubiquinone [74] (Table S8).

Based on the KEGG annotation results, 12 enzymes were identified to be involved in steroid biosynthesis (Table S8). In particular, we identified a single-copy gene encoding lanosterol synthase (LSS) (Gene ID: A3811; EC No.: 1.14.14.17), which synthesizes lanosterol as a squalene or oxidosqualene cyclase family enzyme, a common triterpenoid and cyclic intermediate of steroids [75]. Synthesis of LSS was found in other Basidiomycetes [17,76,77].

For the NRPS-like, two gene clusters (22 genes) related to NRPS-like synthesis were found in the genome. Non-ribosomal peptide synthetase-like has a wide range of biological activities and pharmacological properties, including antibiotics, cytotoxins, immunosuppressants, and siderophores [78]. The NRPS genes predicted in the genome are listed in Table S8.

In addition, gene clusters related to the synthesis of betalactone were also found in the genome, and the numbers were one. It has been well known that betalactone is an antiviral heterocyclic compound [79].

The analysis was not sufficiently extensive, notwithstanding our predictions and hypotheses about the possible secondary metabolites contained in *N. aurantialba*. Kuhnert et al. identified and analyzed biosynthetic gene clusters of hypoxylaceae species based on blastp using Geneious software (v. 9.1.8) [80]. We can use this method to compare the secondary metabolite synthetic gene cluster of *N. aurantialba* to that of other basidiomycetes, create a secondary metabolite-based phylogenetic tree, and draw a schematic structure to gain insight into the mechanism of chemical interaction between basidiomycetes, secondary metabolites, and their environment in future work.

### 3.7. Synthesis of Polysaccharides

Polysaccharides are the main active substances found in *N. aurantialba*, which are generally divided into exopolysaccharides (EPS), cell wall polysaccharides (CWPS), and other polysaccharides (OPS). Studies have found that *N. aurantialba* polysaccharides exert their biological activities through apoptosis, mitogen-activated protein kinase (MAPK), and nuclear factor kappa B (NF-κB) signaling pathways [5].

#### 3.7.1. EPS

*N. aurantialba* was shown to have the ability to produce high-yielding EPS in a previous study, but the mechanism of synthesis was unclear [35].

The synthesis of exopolysaccharide (EPS) by Basidiomycetes is generally divided into three steps: the synthesis of nucleotide-activated sugars, the attachment of sugar chains, and the extracellular export of polysaccharides [81]. Based on the KEGG annotation, a possible synthetic mechanism for EPS synthesis by *N. aurantialba* is discussed from these three aspects.

##### Synthesis of Nucleotide-Activated Sugars

The metabolic pathways of synthetic sugar nucleotides contained in the metabolism of *N. aurantialba* are shown in Figures S7 and S8, Table S9, and a total of 13 enzymes encoded by 15 key genes are involved in nucleotide-activated sugar synthesis. Most of these genes have been identified in medicinal food fungi (*G. lucidum*, *Cordyceps*, and *H. erinaceus*) that have been shown to affect nucleoside sugar synthesis. Furthermore, genes related to the synthesis of guanosine diphosphate (GDP)-mannose, UDP-xylose, and UDP-glucuronic acid were identified, indicating that the monosaccharide components of EPS should include mannose, xylose, and glucuronic acid. This result was consistent with previous experiments in which the monosaccharide fractions were measured [35].

##### Linking and Modification of Sugar Chains

In addition to the synthesis of EPS, nucleoside sugars can also synthesize other glycosylated substances, such as glycoproteins, glycolipids, and saponins. Therefore, glycosyltransferases and glycoside hydrolases are important for the synthesis of polysaccharides [51]. Glycosyl transferases are mainly responsible for the sugar chains. Linked to determine the type of polysaccharide, GHs are responsible for the role of transglycosides in polysaccharide modification [81].

##### Extracellular Export of Polysaccharides

There have not been complete research data on the transmembrane transport of EPS in fungi, but according to the research conducted in bacteria, the mechanism of EPS assembly and export may be relatively conservative, which usually follows Wzy-dependent or adenosine triphosphate (ATP)-binding cassette (ABC)-dependent pathways [81]. The genes that may be involved in polysaccharide transport are listed in Table S10 [81].

#### 3.7.2. CWPS

The cell walls of fungi are usually chitin and glucan. Chen et al. found that the component of cell wall polysaccharide of *T. fuciformis* was achitin-glucan complex [82]. As shown in Table S9, we predicted 21 genes (dextran 11 and chitin 10) that may be related to cell wall synthesis in the KEGG database.

#### 3.7.3. OPS

We also found 260 genes associated with other polysaccharide syntheses (N-glycan, mannose type O-glycan, and others) (Table S9).

### 3.8. Biosynthesis of Bioactive Proteins, Vitamins B, Amino Acids, and Unsaturated Fatty Acids

Bioactive proteins, vitamin B, amino acids, and unsaturated fatty acids play an important role in human health, and mushrooms are also an important source of these active substances [83].

As shown in Table S11, 20 genes related to bioactive proteins (two laccases, 14 ribonucleases, and four lectins), 89 genes related to vitamin, and seven genes related to unsaturated fatty acids in *N. aurantialba* were annotated.

Amino acid is one of the main reasons why mushrooms have a pleasant taste [76]. Genes involved in mushroom amino acid metabolism were predicted in *N. aurantialba* NX-20, wherein 32 genes were involved in glycine, serine, and threonine metabolism (sweet amino acids), and 29 genes were involved in alanine, aspartate, and glutamate metabolism (umami amino acids) (Table S11).

### 3.9. Comparison with Other Basidiomycete Genomes

#### 3.9.1. Gene Family, Core-Pan, and Phylogenetic Analysis

**Figure 3:**
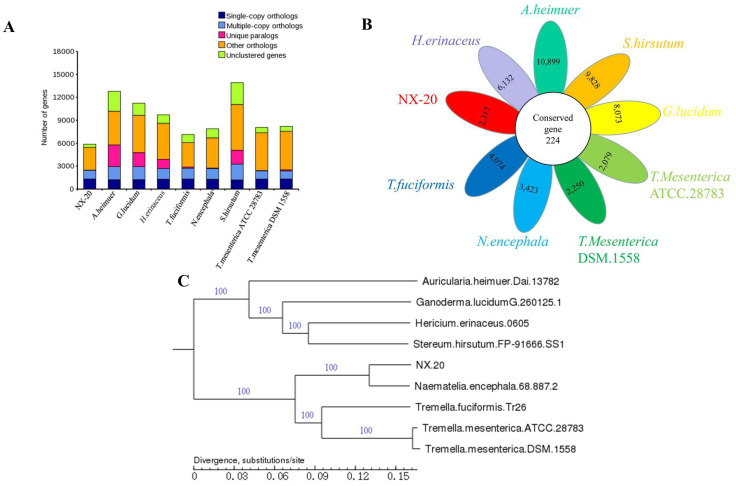
Comparative genomics analysis. (**A**) Gene family (Single-Copy Orthologs, the number of single-copy homologous genes in the species common gene families; Multiple-Copy Orthologs, the number of multiple-copy homologous genes in the species common gene families; Unique Paralogs, genes in specific gene families; Other Orthologs, other genes; Unclustered Genes, genes that have not been clustered into any families); (**B**) conserved and specific gene counts (each ellipse represents a strain, and the numbers in the ellipses are specific genes. In addition, the central white circle represents conserved genes among the nine strains); (**C**) maximum likelihood phylogenetic tree.

A gene family is a collection of related genes that result from the duplication of a single initial gene [84]. The statistics of gene family numbers were obtained according to the cluster of orthologous groups based on protein sequences of strains (Figure 3A). *N. aurantialba* has a lower number of genes (genes number, genes in families, unclustered genes, family number, and unique families) than other strains, according to gene family analysis. Moreover, the number of genes, genes in families, unclustered genes, families, and unique families in yeast-like basidiomycetes was lower than in filamentous basidiomycetes. Gene gain or loss events may occur in the evolution of basidiomycetes, and gene loss events are more common than gene evolution events in the evolution of yeast-like basidiomycetes [85,86]. Thus, gene family analysis indicates that *N. aurantialba* has fewer duplications and more losses, resulting in fewer genes overall than the other three yeast-like basidiomycetes.

The CD-HIT rapid clustering of similar protein software was used to analyze the core pan of *N. aurantialba* with several typical basidiomycetes. Previous studies on the core-pan analysis were mostly done at the genus or family level because the only strains in the same genus or even family as *N. aurantialba* that have been sequenced are *N. encephala*, so the eight most typical basidiomycetes were selected and core-pan analysis was performed at the class level to investigate functional differences and similarities among the strains [87,88,89].

We identified 55,120 pan genes (all the genes in nine fungi) in the nine analyzed strains containing 224 conserved genes (the homologous genes that were present in all samples) and 54,896 other genes (Figure 3B), wherein *A. heimuer* had the most species-specific genes (*n* = 10,899), followed by *S. hirsutum* (*n* = 9828), *G. lucidum* (*n* = 8073), *H. erinaceus* (*n* = 6132), NX-20 (*n* = 2317), *T. fuciformis* (*n* = 4074), *N. encephala* (*n* = 3423), and *T. mesenterica* (*n* = 2079 and 2250).

The results of phylogenetic analysis are shown in Figure 3C; *N. aurantialba* NX-20 had the greatest taxonomically related to *N. encephala*, followed by *T. fuciformis* and then *T. mesenterica.*

#### 3.9.2. Genomic Synteny

**Figure 4:**
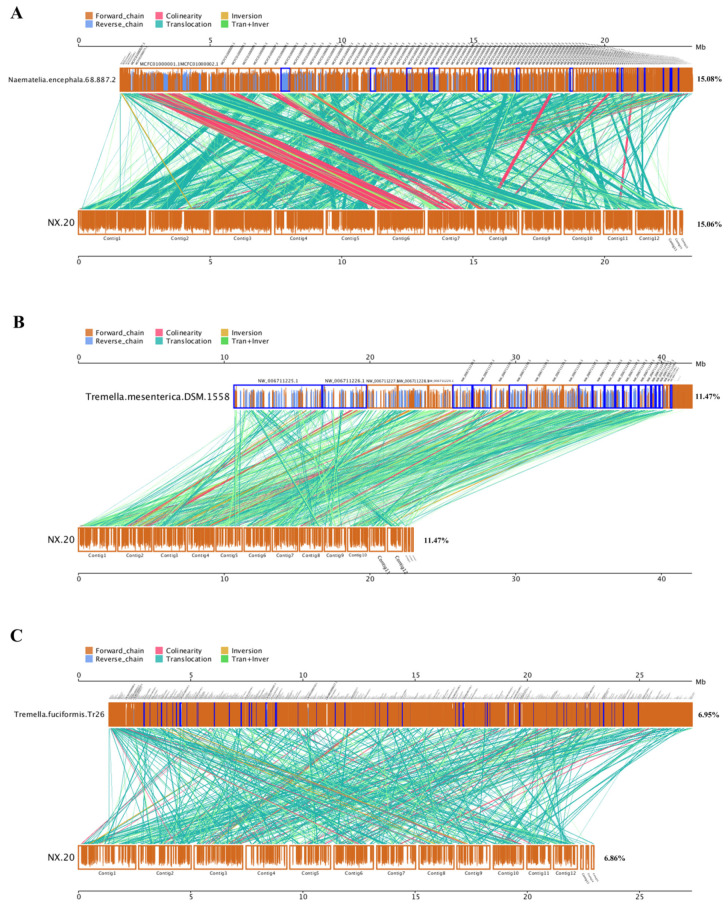
Synteny of *N. aurantialba* NX-20 with N. encephala 68-887.2 (**A**), *T. mesenterica* DSM 1558 (**B**), and *Tremella fuciformis* Tr26 (**C**). The upper axis indicates the genome measured, and the lower axis indicates the reference sequence genome. The forward and reverse strands are represented by yellow boxes and blue boxes, respectively. The height of the filled color in the box indicates the similarity of the alignment, and full filling indicates 100% similarity. The color of the linked graph between the upper and lower axes indicates the alignment type: Collinear, syntenic alignment; Translocation, translocation alignment; Inversion, inverted alignment; Tran + inver, alignment of translocations and inversions.

Figure 4 shows the collinearity of genes in the whole genome sequences of *N. aurantialba* NX-20 and three mushrooms belonging to the order Tremellales.

We found that NX-20 had the highest collinearity with *N. encephala* 68-887.2 (15.08%), followed by *T. mesenterica* DSM 1558 (11.47%) and *T. fuciformis* tr26 (6.95%). This result further showed the closer proximity of NX-20 to *N. encephala* 68-887.2, and grouping NX-20 into the genus *Naematelia* rather than Tremella might be a more appropriate choice.

## 4. Conclusions

Genome sequencing and functional annotation provide valuable information for determining the potential function and gene expression mechanism, which can be used to provide a theoretical basis for *N. aurantialba* breeding, high-yield cultivation, and the construction of molecular biology platforms. This study showed that *N. aurantialba* NX-20 has the potential to be used to synthesize a variety of secondary metabolites, especially highly enriched polysaccharides and terpenoid biosynthesis genes, providing important insights into the biological properties of the medicinal food fungus *N. aurantialba* through whole-genome sequencing, including its gene regulatory network, growth characteristics, and biosynthetic pathways of active compounds, among others. In addition, this study is expected to provide fundamental information for developing mushroom genomes and genetic resources to elucidate the molecular mechanisms underlying the synthesis of multiple secondary metabolites in pharmaceutical edible mushrooms. To the best of our knowledge, this genome-wide assembly and annotation data represent the first genome-scale assembly of this species.

Although the relevant information was incomplete, the analysis of the *N. aurantialba* genome in this study fills the gap in genomic information on *N. aurantialba* and will lay a theoretical foundation for future research on the biosynthesis of active compounds, promoting the application of *N. aurantialba* in the field of drug research and functional food development.

## Figures and Tables

**Table 1:** T Statistics of *N. aurantialba* NX-20 genome assembly and gene prediction.

Feature	Value
Genome assembly	
Contigs number	15
Max length (bp)	2,546,384
N50 length (bp)	1,814,705
Total length (bp)	20,998,359
GC (%)	56.42
Gene prediction	
Gene number	5860
Gene total length (bp)	8,989,977
Gene average length (bp)	1534
Gene length/Genome (%)	42.81

**Table 2:** Assembly summary statistics compared to other mushrooms of Tremellales.

Species	NCBI BioProject	Total Length (Mb)	GC%	Contigs	N50 Length (bp)	Completeb ^a^	Fragmented	Missing
*T. fuciformis* Tr26	PRJNA281519	23.6356	57.0	3502	18,448	92.4%	1.4%	6.2%
*T. mesenterica* DSM 1558	PRJNA225529	28.6399	46.8	484	123,767	92.0%	1.4%	6.6%
*T. mesenterica* ATCC 28783	PRJNA207298	27.1109	41.3	1019	73,463	90.6%	2.4%	7.0%
*N. encephala* 68-887.2	PRJNA330699	19.7863	49.3	151	209,500	85.5%	3.4%	11.1%
*N. aurantialba* NX-20	PRJNA772294	20.9984	56.4	15	1,825,336	93.1%	2.4%	4.5%

Note: ^a^ number of BUSCO proteins (percent of total BUSCOs).

**Table 3:** Statistical results of repeat sequences in the *N. aurantialba* NX-20 genome.

Repeat Type	Type	Number of Elements	Length Occupied (bp)	Repeat Size (bp)	Percentage of Genome (%)
Interspersed repeat	SINE	9	1030	-	0.0049
	LINEs	395	39,539	-	0.1883
	LTR elements	643	115,566	-	0.5504
	DNA elements	418	39,329	-	0.1873
	RC	68	8542	-	0.0407
	Unknown	16	1593	-	0.0076
Tandem repeat	TR	12,449	583,229	1~982	2.7775
	Microsatellite DNA	1448	91,405	2~6	0.4353
	Minisatellite DNA	9096	453,057	10~60	2.1576

Note: -, not detected.

**Table 4:** Statistical results of noncoding RNAs in the *N. aurantialba* NX-20 genome.

Type	Number of Elements	Total Length (bp)	Average Length (bp)	Percentage in Genome (%)
tRNA	44	3925	89	0.01869
5s_rRNA	9	1034	115	0.00599
5.8s_rRNA	0	0	0	0
18s_rRNA	1	1802	1802	0.02294
28s_rRNA	1	3492	3492	0.05030
sRNA	0	0	0	0
snRNA	7	677	96	0.00322
miRNA	0	0	0	0

## Data Availability

Genome sequencing of N. aurantialba-20 generated for this study have been submitted to the NCBI (https://www.ncbi.nlm.nih.gov, accessed on: 1 November 2021). BioProject: PRJNA772294 and BioSample: SAMN22141859.

## Supplementary Materials

The following are available online at https://www.mdpi.com/article/10.3390/jof8010006/s1, Figure S1: Summarizes of the annotations of N. aurantialba; Figure S2: The KOG function classification of proteins in N. aurantialba; Figure S3: Comparative genomics analysis of KOG annotations; Figure S4: The GO function annotation of N. aurantialba; Figure S5: The KEGG function annotation of N. aurantialba; Figure S6: Terpenoid biosynthesis pathway of N. aurantialba; Figure S7: Amino sugar and nucleotide sugar metabolic pathway in N. aurantialba; Figure S8: Putative nucleoside sugar biosynthetic pathway of N. aurantialba; Table S1: Basidiomycetes and the origin of their genomes used in the study; Table S2: Comparative genomics analysis of Repeat sequence and Noncoding RNA; Table S3: Summarizes of the annotations of Naematelia aurantialba; Table S4: Comparative genomics analysis of KOG, GO, and KEGG annotations; Table S5: Distribution of CAZyme in Naematelia aurantialba and the other 18 basidiomycetes; Table S6: P450 genes and subfamilies in Naematelia aurantialba; Table S7: The putative enzyme-coding genes involved in biosynthesis of secondary metabolites; Table S8: The putative genes involved in secondary metabolism of Naematelia aurantialba; Table S9: The putative genes involved in polysaccharides biosynthesis of Naematelia aurantialba; Table S10: Putative genes involved in ABC-dependent pathways; Table S11: The putative genes involved in biosynthesis of bioactive proteins, vitamins B, amino acids, and unsaturated fatty acids of Naematelia aurantialba.
