# Supplementary material for: Whole Genome Sequencing and Annotation of Naematelia aurantialba (Basidiomycota, Edible-Medicinal Fungi)
Source: J Fungi (Basel). 2021 Dec 22;8(1):6. doi: 10.3390/jof8010006 (PMC8777972; doi:10.3390/jof8010006)
Supplement: Supplementary file 1 [file jof-08-00006-s001.zip › jof-1481592-supplementary/Revised-Supplementary material.pdf]

Whole genome sequencing and genome annotation of *Naematelia aurantialba* (Basidiomycota，Edible-medicinal fungi)

Tao Sun[a], Yixuan Zhang[a],Hao Jiang[a], Kai Yang[a], Shiyu Wang[a], Rui Wang[a], Sha Li[a], Peng Lei[a*], Hong Xu[a], Yibin Qiu[b], Dafeng Sun[c].

[a] *State Key Laboratory of Materials-Oriented Chemical Engineering, College of Food Science and Light Industry, Nanjing Tech University, Nanjing 211816, China.*

[b] *College of Light Industry and Food Engineering, Nanjing Forestry University, Nanjing 210037, China*

[c] *Kunming Edible Fungi Institute of All China Federation of Supply and Marketing Cooperatives, Kunming 650032, Yunnan, China*

* Corresponding authors:

Peng Lei, E-mail address: lei-peng@njtech.edu.cn

Tel: +86 18761681790

Figure S1

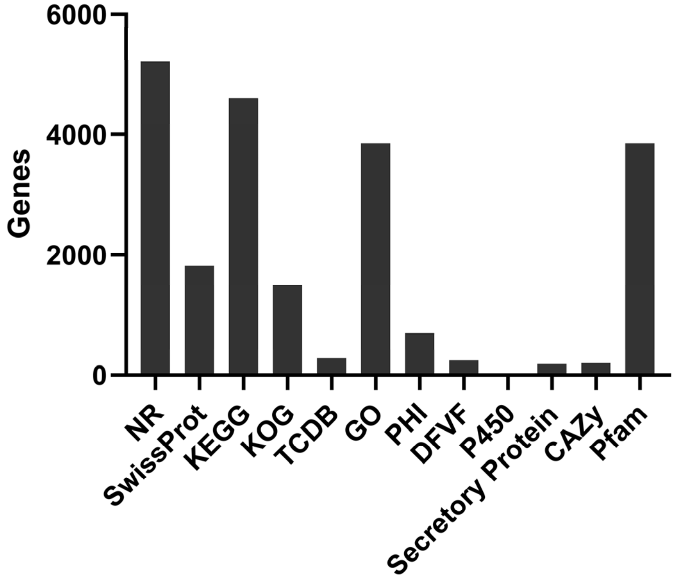

Figure S1. Summarizes of the annotations of *N. aurantialba*.

Figure S2

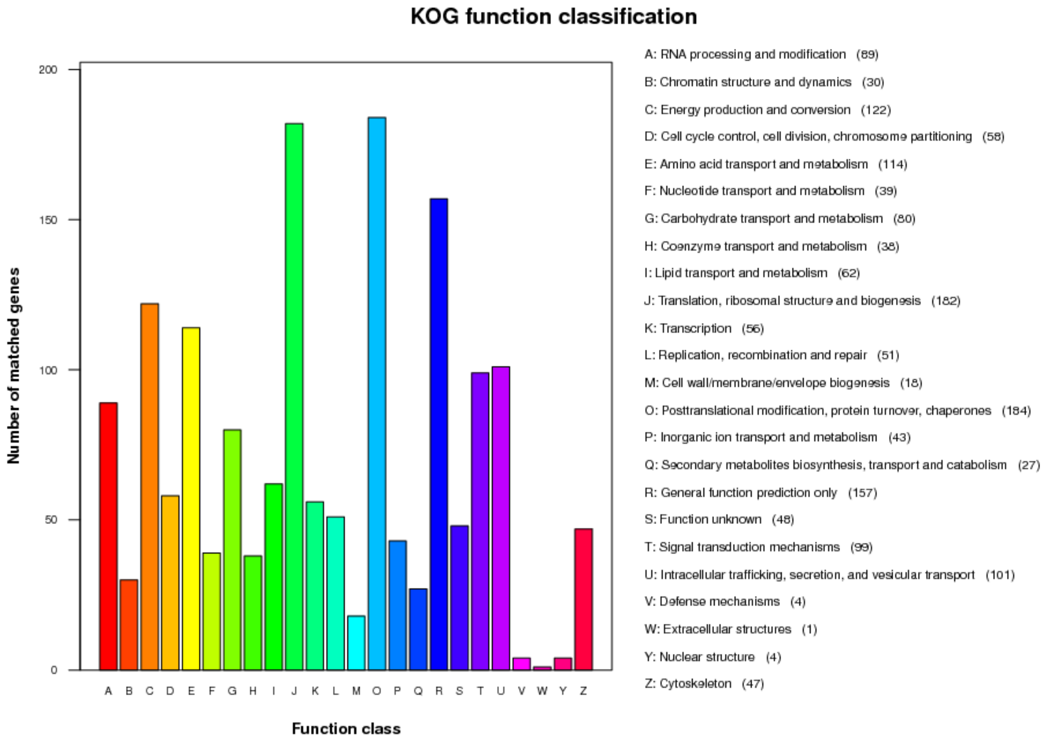

Figure S2. The KOG function classification of proteins in *N. aurantialba*.

Figure S3

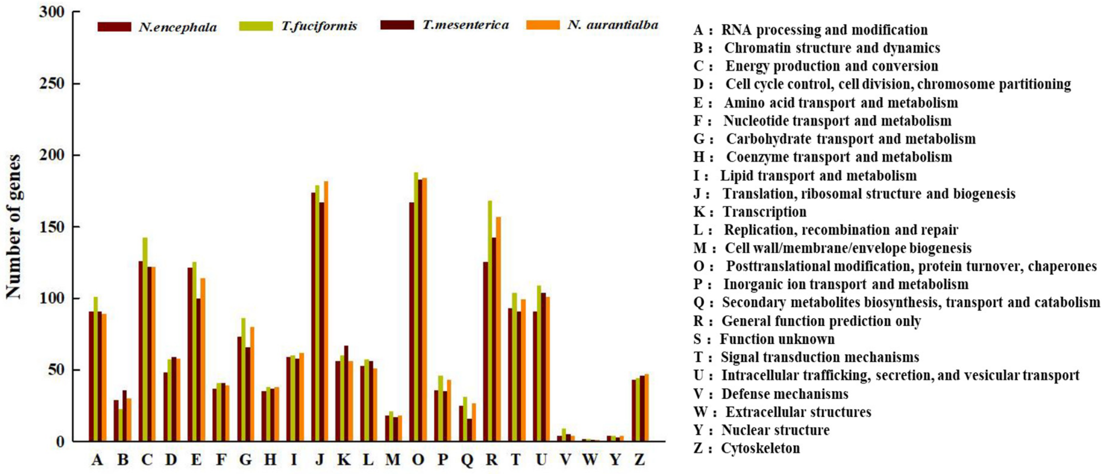

Figure S3. Comparative genomics analysis of KOG annotations.

Figure S4

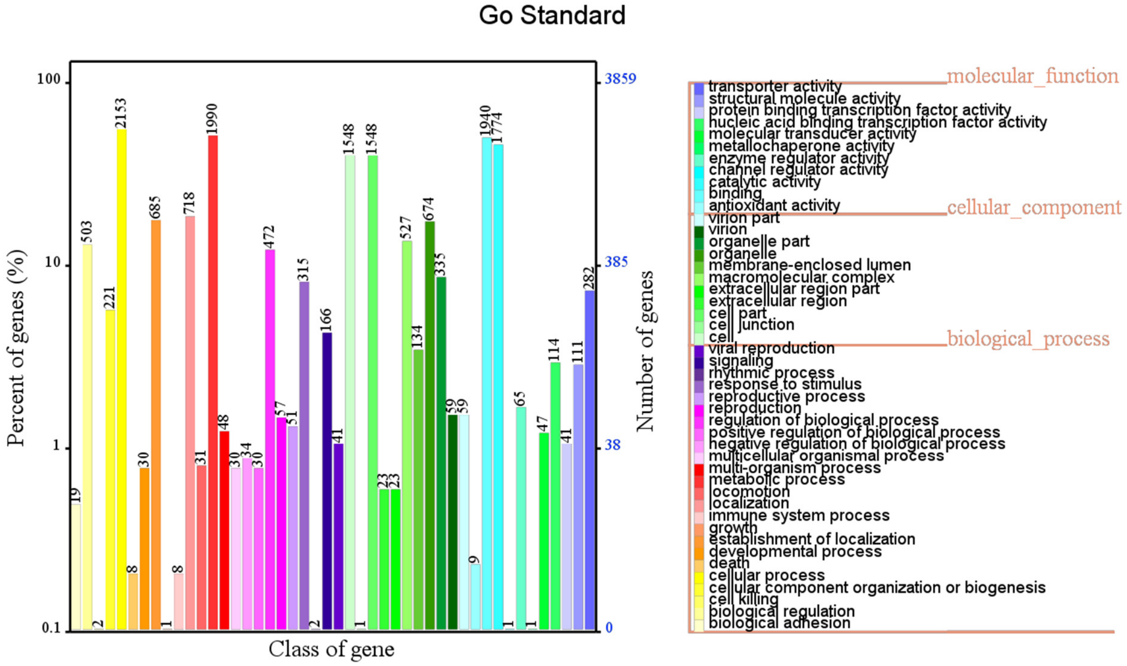

Figure S4. The GO function annotation of *N. aurantialba*.

Figure S5

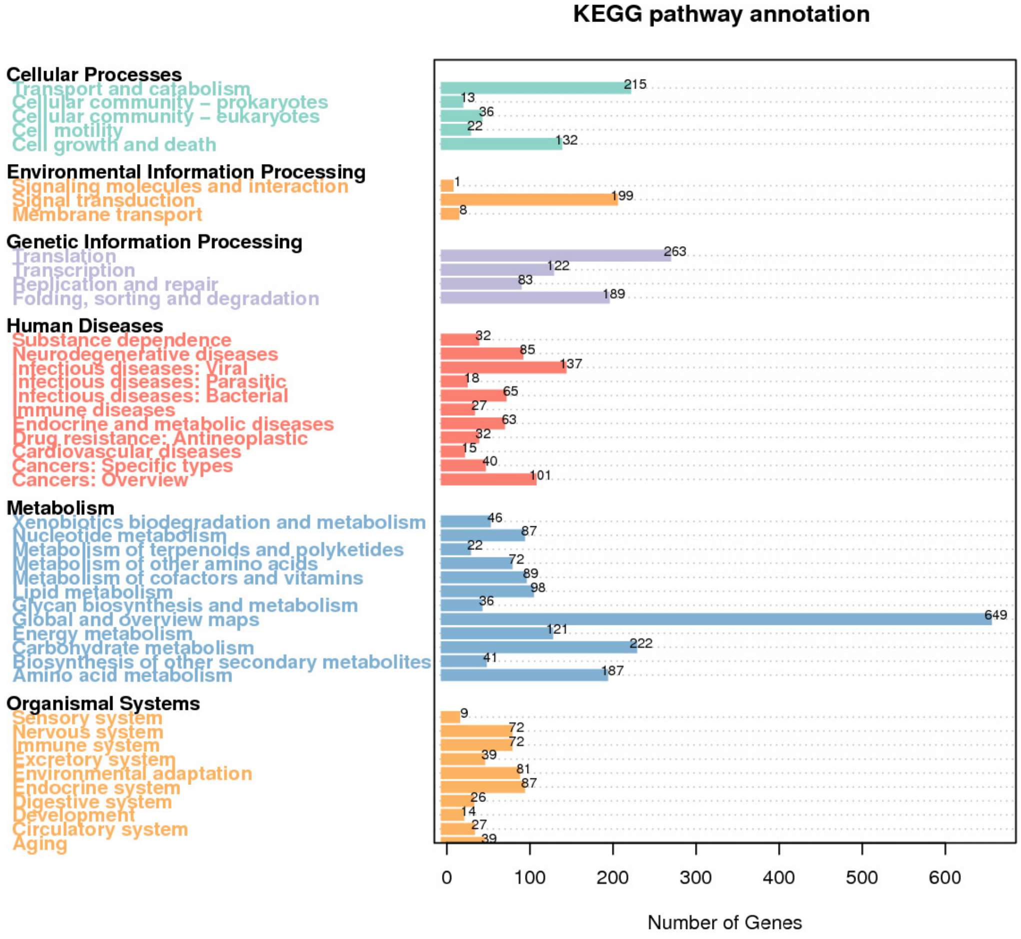

Figure S5. The KEGG function annotation of *N. aurantialba*.

Figure S6

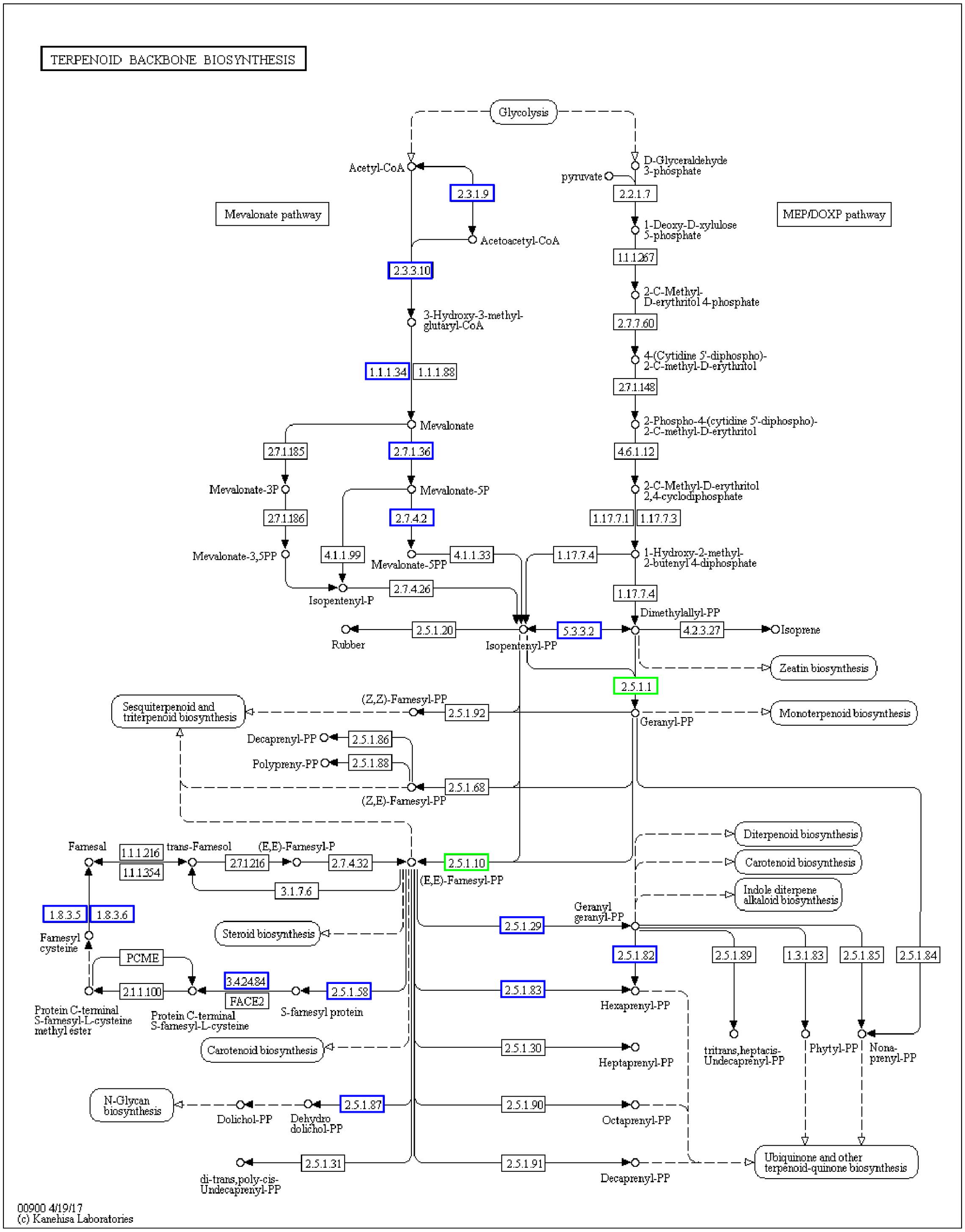

Figure S6. Terpenoid biosynthesis pathway of *N. aurantialba*.

Note: The different colors of the boxes indicate the number of corresponding genes. Blue represents 1 gene and green represents 2 genes, while white means not.

Figure S7

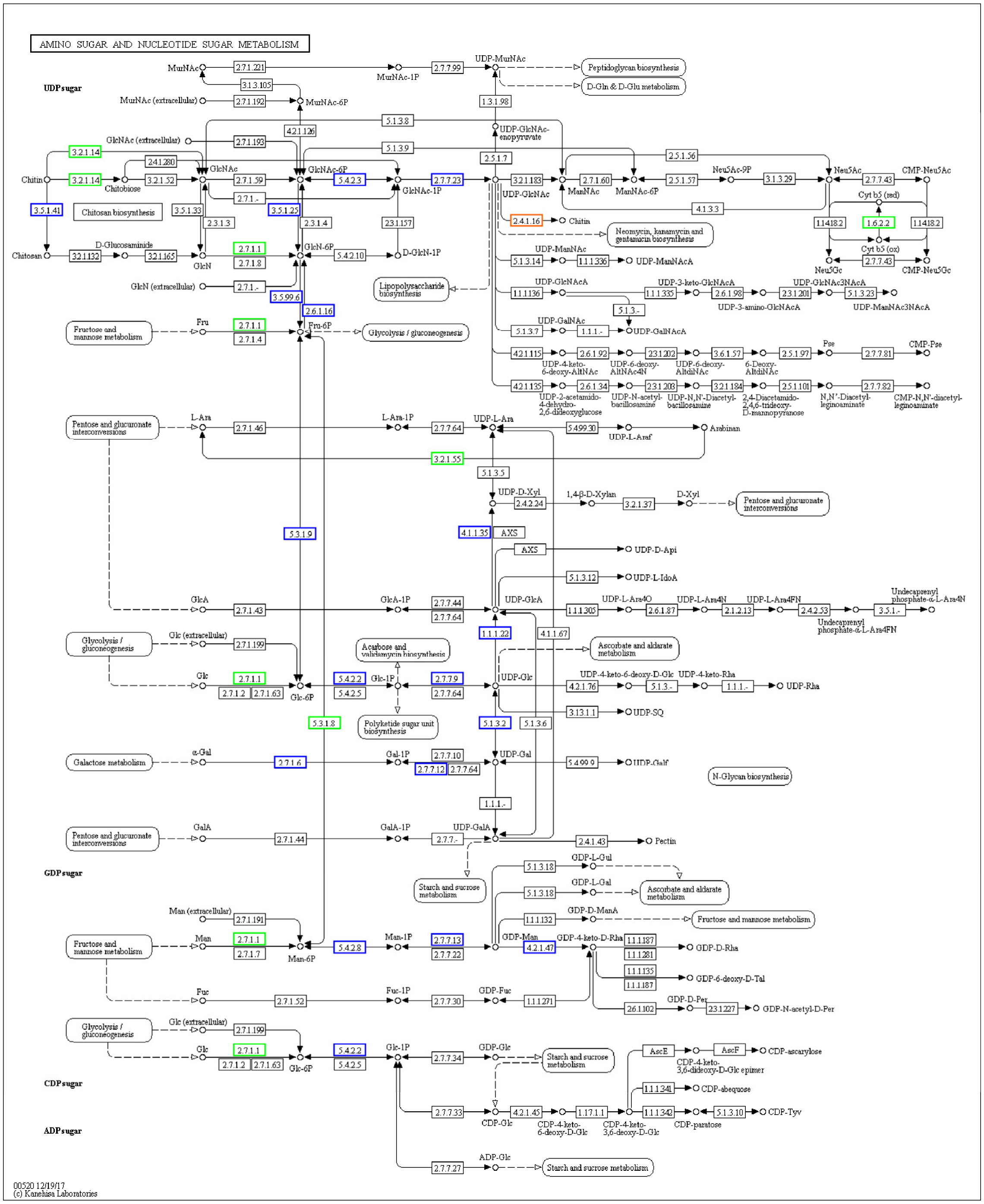

Figure S7. Amino sugar and nucleotide sugar metabolic pathway in *N. aurantialba*.

Note: The different colors of the boxes indicate the number of corresponding genes. Blue represents 1 gene, green represents 2 genes, yellow 4-9 genes, and red represents more than 10 genes, while white means not.

Figure S8

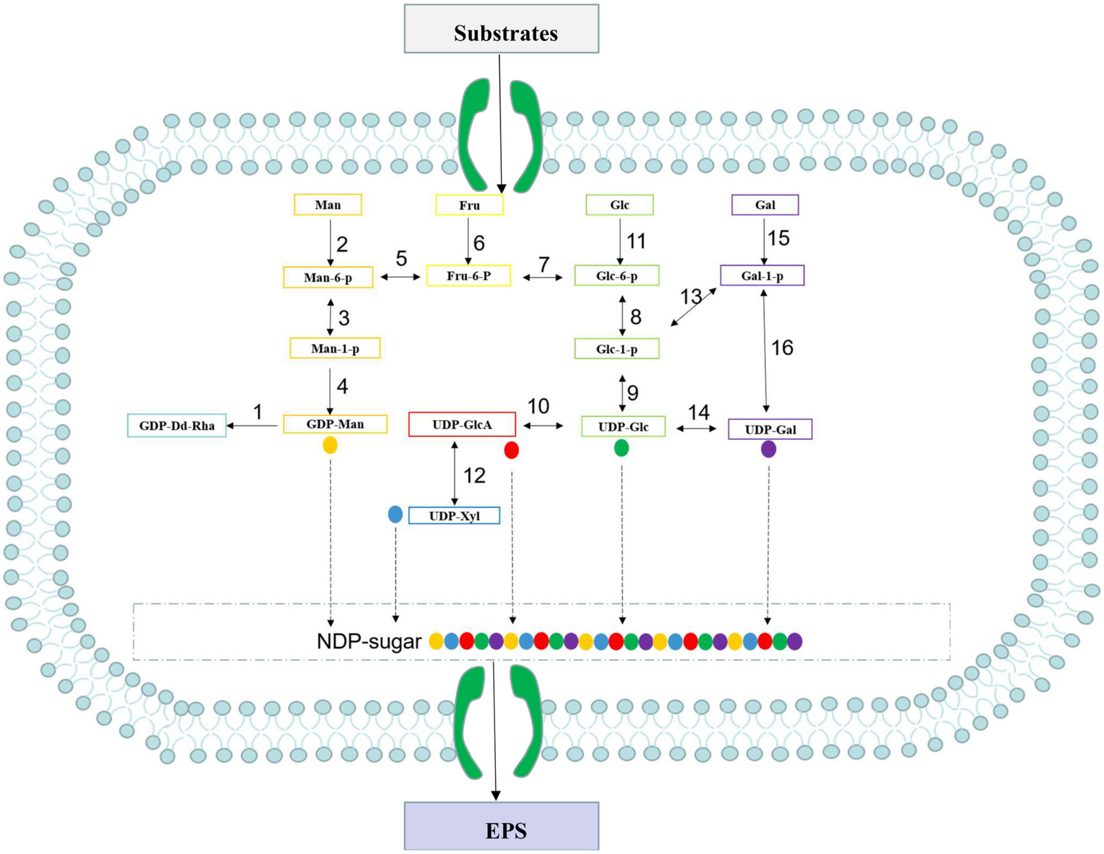

Figure S8. Putative nucleoside sugar biosynthetic pathway of *N. aurantialba*.

Notes: Glucose, galactose, mannose, and fucose are able to synthesize nucleoside sugars via metabolic pathways and the enzymes required to catalyze these reactions.

1. EC [4.2.1.47]. 2,6,11. EC [2.7.1.1]. 3. EC [5.4.2.8]. 4. EC [2.7.7.13]. 5. EC [5.3.1.8]. 7. EC [5.3.1.9]. 8. EC [5.4.2.2]. 9. EC [2.7.7.9]. 10. EC [1.1.1.22]. 12. EC [4.1.1.35]. 13,16. EC [2.7.7.12]. 14. [5.1.3.2]. 15. EC [2.7.1.6].

Abbreviations:

EPS, Exopolysaccharides; Fru, fructose; Fru-6-P, fructose-1,6-bisphosphate; Gal, galactose; Gal-1-p, alpha-galactose-1-phosphate; GDP-Dd-Rha, GDP-4-dehydro-6-deoxy-mannose; GDP-Man, GDP-mannose; Glc, glucose; Glc-1-p, glucose-1-phosphate; Glc-6-p, glucose-6-phosphate; Man, mannose; Man-1-p, mannose-1-phosphate; Man-6-p, mannose-6-phosphate; UDP-Gal, UDP-alpha-galactose; UDP-Glc, UDP-glucose; UDP-GlcA, UDP-glucuronate; UDP-Xyl, UDP-xylose.
